# GARNET – gene set analysis with exploration of annotation relations

**DOI:** 10.1186/1471-2105-12-S1-S25

**Published:** 2011-02-15

**Authors:** Kyoohyoung Rho, Bumjin Kim, Youngjun Jang, Sanghyun Lee, Taejeong Bae, Jihae Seo, Chaehwa Seo, Jihyun Lee, Hyunjung Kang, Ungsik Yu, Sunghoon Kim, Sanghyuk Lee, Wan Kyu Kim

**Affiliations:** 1 Information Center for Bio-Pharmacological Network, Seoul National University, Suwon 443-270 , Korea; 2 Division of Molecular Life Sciences, Ewha Womans University, Seoul 120-750, Korea; 3 Ewha Research Center for Systems Biology (ERCSB), Ewha Womans University, Seoul 120-750, Korea; 4 Center for Medicinal Protein Network and Systems Biology, College of Pharmacy, Seoul National University, Seoul 151-742, Korea; 5 Cancer and Diabetes Institute, Gachon University of Medicine and Science, Incheon, 406-840, Korea

## Abstract

**Background:**

Gene set analysis is a powerful method of deducing biological meaning for an a priori defined set of genes. Numerous tools have been developed to test statistical enrichment or depletion in specific pathways or gene ontology (GO) terms. Major difficulties towards biological interpretation are integrating diverse types of annotation categories and exploring the relationships between annotation terms of similar information.

**Results:**

GARNET (Gene Annotation Relationship NEtwork Tools) is an integrative platform for gene set analysis with many novel features. It includes tools for retrieval of genes from annotation database, statistical analysis & visualization of annotation relationships, and managing gene sets. In an effort to allow access to a full spectrum of amassed biological knowledge, we have integrated a variety of annotation data that include the GO, domain, disease, drug, chromosomal location, and custom-defined annotations. Diverse types of molecular networks (pathways, transcription and microRNA regulations, protein-protein interaction) are also included. The pair-wise relationship between annotation gene sets was calculated using kappa statistics. GARNET consists of three modules - *gene set manager*, *gene set analysis* and *gene set retrieval*, which are tightly integrated to provide virtually automatic analysis for gene sets. A dedicated viewer for annotation network has been developed to facilitate exploration of the related annotations.

**Conclusions:**

GARNET (gene annotation relationship network tools) is an integrative platform for diverse types of gene set analysis, where complex relationships among gene annotations can be easily explored with an intuitive network visualization tool (http://garnet.isysbio.org/ or http://ercsb.ewha.ac.kr/garnet/).

## Background

Omics studies usually yield a number of gene lists e.g. differentially expressed genes (DEGs). Typically, a statistical test of enrichment or depletion is performed for an a priori defined set of genes (usually from clustering of microarray data) or gene annotations. This approach has been successfully applied for diverse subjects including gene ontology (GO), signalling and metabolic pathways, and identification of regulatory elements such as transcription factors and microRNAs. However, biological interpretation of gene lists is still a challenge for many biologists because there is no ‘golden standard method’ established yet. Numerous annotation DBs and tools have been developed for biological interpretation of experimental gene lists including but not limited to GSEA [1], DAVID [2], Gazer [3], FatiGO+ [4], g:Profiler [5], WebGestalt [6] Lists2Networks [7] and GOAL [8]. A comprehensive list of 68 GSA web tools is recently reviewed by Huang *et al*[9] as well as several important points to consider in using such tools. As Huang and colleagues suggests, each tool has its own strength and limitations in terms of statistical method, coverage of gene annotation types and user interface [9].

One important issue in the field is the growing complexity of annotation data themselves. The benefit of gene set analysis (GSA) mainly comes from the power to summarize hundreds or even thousands of genes into a smaller number of enriched biological themes e.g. GO term or pathways, allowing simplified interpretation of high-throughput experiments. However, the analytic complexity of GSA is getting beyond its benefits because of the rapid increase of gene annotations e.g. a few dozens of genes can be enriched in a hundred or more annotation terms. The number of GO terms is already more than the number of genes in a human genome and the situation is getting similar with other types of annotation like pathways, the regulatory targets of TFs and miRNA, disease-associated genes and chromosomal locations even without considering their combinations [10]. Increasingly, omics data continue to be sources of new annotations e.g. cancer signature genes from microarray [11] and disease-associated genes from GWAS studies [12]. Major difficulties towards meaningful biological interpretation are integrating diverse types of annotations and at the same time, handling the complexities for efficient exploration of annotation relationships.

GARNET (Gene Annotation Relationship Network Tools) is an integrative platform for diverse types of gene set analysis, allowing convenient annotation network navigation. The utility of GARNET is two-fold. One is to facilitate the interpretation of gene sets from high-throughput experiments such as microarray, ChIP-chip (ChIP-Seq) and high-throughput screening. The other is to serve as a framework for meta-analysis of heterogeneous annotations and pre-existing knowledge, which often lead to novel insights undetectable by individual analyses [13]. In an effort to allow access to a full spectrum of amassed biological knowledge, we have integrated a variety of annotation data that include the GO, domain, disease, drug, chromosomal location, and custom-defined annotations. Diverse types of molecular networks (pathways, transcription and microRNA regulations) are also included. To deal with the complexity from a large number of annotations from different categories, a dedicated annotation network viewer has been developed for the visualization of related annotations.

GARNET system consists of three modules – *gene set retrieval tool*, *gene set manager tool*, and *gene set analysis tool*, which are tightly integrated to allow access, manipulation and statistical analysis of pre-compiled gene annotations and user-defined gene lists. The relationship between annotation terms is calculated using Cohen’s kappa statistic. Kappa is less sensitive to the gene set size than other P-value statistics such as Chi-square, hypergeometric and binomial test because it measures the difference between the observed and the expected agreement between two annotation terms.

## Construction and content

### System overview

**Figure 1:**
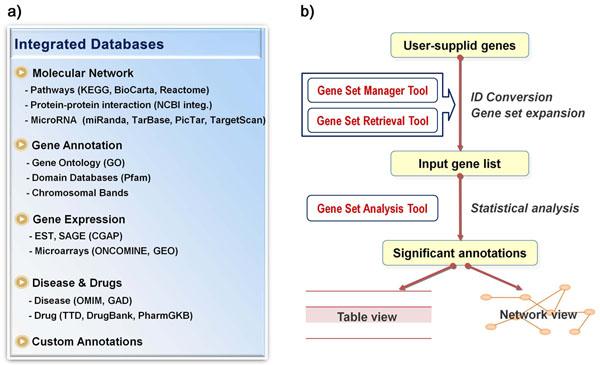
**GARNET system overview**. a) The annotation categories and types integrated in the GARNET system. b) The schematic overview of the workflow. The three main modules in the GARNET system are *Gene Set Manager Tool*, *Retrieval Tool* and *Analysis Tool*. The user-supplied genes are converted to standard gene IDs (Entrez Gene ID) by *Manager Tool*. A Gene set can be expanded via biological networks in *Retrieval Tool*. *Analysis Tool* performs gene set analysis (GSA) against the GARNET annotations selected by the user.

Considering highly exploratory nature of gene set analysis, GARNET is designed to navigate different layers of gene annotations in a convenient and integrated environment, equipped with ID conversion module, an interactive network viewer and import/export of any gene sets. GARNET contains four major groups of gene annotation information – molecular network, gene function, gene expression, and disease & drugs (Figure 1a). Molecular network data include pathways, protein-protein interactions, and microRNA targets from several different sources. We also included gene annotation data such as gene ontology, protein domain and chromosomal location as well as differentially expressed genes (DEGs) from gene expression data. Gene-disease and gene-drug associations are also complied from relevant sources [14]. Further, any user-defined gene annotation data can be uploaded and analyzed against all the annotations in the system. The detailed scope of annotation data is provided in Table 1 and on-line.

**Table 1:** Summary of the annotation categories and types in the GARNET system

Category	Annotation DB	Number of Annotations	Number of Unique Genes	Version	Update Date
**Molecular Network**	KEGG	199	5193	Release 54.0+/	2010.05.10
	BioCarta	314	1383		2010.04.06
	Protein-Protein Interactions (PPI)	5829	7631	NCBI integrated DB	2010.05.17
	miRBase	711	34525	5.0	2009.09.29
	TarBase	106	394	V5	2008.06
	TargetScan	162	7930	4.2	
	PicTar-4way	178	9152	hg17, Build 35	
	PicTar-5way	130	3455	hg17, Build 35	
**Genome Annotation**	Gene Ontology (GO)	30479	18242		2010.05.20
	Pfam domains	3922	16409	24.0	2009.10.07
	Chromosomal Bands	3078	32920		2010.05.23
**Gene Expression**	Tissue EST	51	13367		2010.04.06
	Tissue SAGE	32	8528		2010.04.06
	Tissue Microarray	32	10769		
	Cancer Microarray	25	13619		
**Disease & Drug**	OMIM	1063	2683		2008.08.27
	GAD	1212	2872		2008.12.26
	DrugBank	591	259		
**Total**		57973	32920		

The GARNET system consists of three main tools – manager, analysis, and retrieval tools. Figure 1(b) shows the workflow of GARNET analysis. Users define gene sets using the manager tool where the set operation is available to combine two gene sets as union, intersection, and subtraction. The analysis tool performs enrichment test for the user-supplied genes in the annotation categories of choice. Multiple test correction is applied by default since there are so many annotation terms. The result of statistical analysis is given in table format where one can access the network view of annotation terms. In addition to the flat table view, GARNET also supports the tree view for hierarchical annotations such as GO and OMIM disease terms. The retrieval tool allows users to access the annotation database to extract genes assigned to any annotation term. Importantly, users may expand their gene list using the molecular networks in the annotation database. This novel feature allows users to investigate the down-stream effects of their genes of interest.

Any gene set analysis requires an ID system. We chose the Entrez geneID as the reference ID system instead of devising our own. This alleviates the maintenance (update & expansion) problem significantly since most annotation databases provide the cross-reference to the Entrez geneID. GARNET supports most major gene IDs including Entrez Gene ID, Ensembl Ids (Gene, Transcript), GenBank, RefSeq, UniProt and microarray probe IDs (Affymatrix, Illumina). The detailed list of supported ID types is listed in Table 2. Even though we support diverse ID types for input, all subsequent analyses are carried out at the gene level, not protein or transcript level.

**Table 2:** List of supported ID types in GARNET

Category	ID Type	Examples
**Gene ID**	Entrez Gene	1457, 2002, 1950
	Ensembl Gene	ENSG00000101266
	Ensembl Transcript	ENST00000361797
	GenBank Accession	AB451279, AI628974
	GenBank ID	19769225, 4665774
	RefSeq	NM_130786, NM_000014
	UniGene	Hs.41, Hs.56
	HGNC Gene Symbol	A1BG, A1CF, A2LD1
	HGNC Gene ID	7, 8, 7645
**Protein ID**	UniProtKB Accession	P62258, Q04917
	UniProtKB ID	1433B_HUMAN
	PIR Accession	S34755, A61235, I38947
**Microarray**	Affymetrix probe ID	212073_at, 210984_x_at
	Illumina probe ID	4210086, 2510484

### Datasets

A comprehensive set of gene annotation data are integrated in the GARNET system (Table 1). The annotations are grouped into four different categories of molecular network, genome annotation, gene expression, and disease & drug. The molecular network category consists of pathways (KEGG [15], BioCarta), protein-protein interactions (PPI) from NCBI and four major miRNA target databases (miRBase [16], TarBase [17], TargetScan [18] and PicTar [19]). The category of genome annotation contains information on gene function (Gene Ontology [20]), protein domain (Pfam [21]) and chromosomal location. Tissue-specific or cancer-related gene expression data are included in the gene expression category. We also collected gene-diesease (OMIM [22], GAD [12]) and gene-drug association (DrugBank [14]) data from relevant sources, that are deposited in the disease & drug category.

### User interface

#### Manager Tool

**Figure 2:**
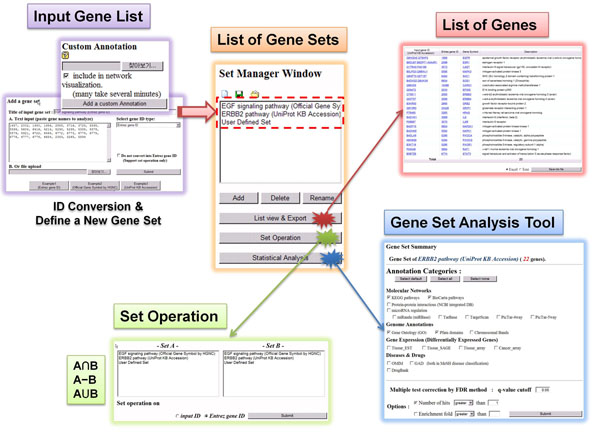
**The gene set manager tool**.

The gene set manager tool (*Manager Tool*) is the main entry point to access all the annotation data and further gene set analysis in GARNET. The overall workflow is shown in Figure 2. In *Manager Tool*, user may create or delete a new gene set by either entering a list of gene IDs or uploading an input file. The input gene IDs are automatically converted to Entrez Gene IDs. All the user-created gene sets are listed up in a separate panel named *Set Manager Window* (dotted red box in Figure 2), where the gene set of interest can be chosen. Users can access the member genes of the chosen gene set or create a new gene set by set operation of existing gene sets e.g. interaction, subtraction, and union. Once gene sets are prepared in the *Manager Tool*, user can proceed to *Analysis Tool* for gene set analysis or *Retrieval Tool* for expanding gene list along the biological networks as described in the later sections.

#### Analysis Tool

**Figure 3:**
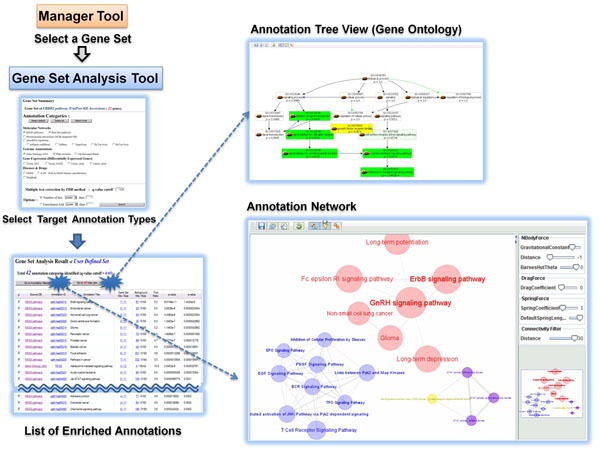
The gene set analysis tool.

*Analysis Tool* implements the core function of statistical over-representation analysis against various types of gene annotations. User selects a target gene set and can perform GSA against multiple types of annotations simultaneously. The overall procedure of GSA is shown in Figure 3. Kappa statistic is used as the enrichment score in GARNET. Multiple test correction is done by Benjamini-Hochberg method. The stringency of GSA is set as q-value<0.05 by default, but the q-value cut-off can be adjusted according to the user’s preference as well as by the number of hit annotations. As a result of GSA, the list of significantly enriched annotations is displayed as table view. Particularly, the relationships among the enriched annotations are presented in an interactive network viewer. The annotation network data are downloadable in standard formats for other visualization tools (e.g. cytoscape) or network clustering softwares (e.g. MCL [23]) for further analysis. It also opens the chance to reveal connections among heterogeneous types of annotations e.g. miRNA targets and cancer signature genes, drug targets and protein. Annotations such as Gene Ontology and MeSH terms have hierarchical structure, which is displayed in tree view as well (top-left in Figure 3).

#### Retrieval Tool

**Figure 4:**
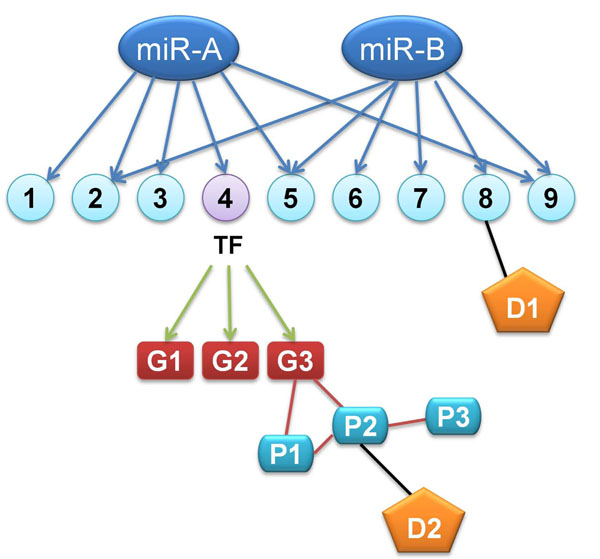
**An example of gene set expansion via various types of biological networks**. The targets of two microRNAs (miR-A and miR-B) can be expanded by transcription factor (TF)-target network (green edge) to include G1~G3. The gene set is further expanded via protein-protein interaction network (red edge) to include P1~P3. D1~P2 and D2-8 represents Drug-target relationship (black edge).

**Figure 5:**
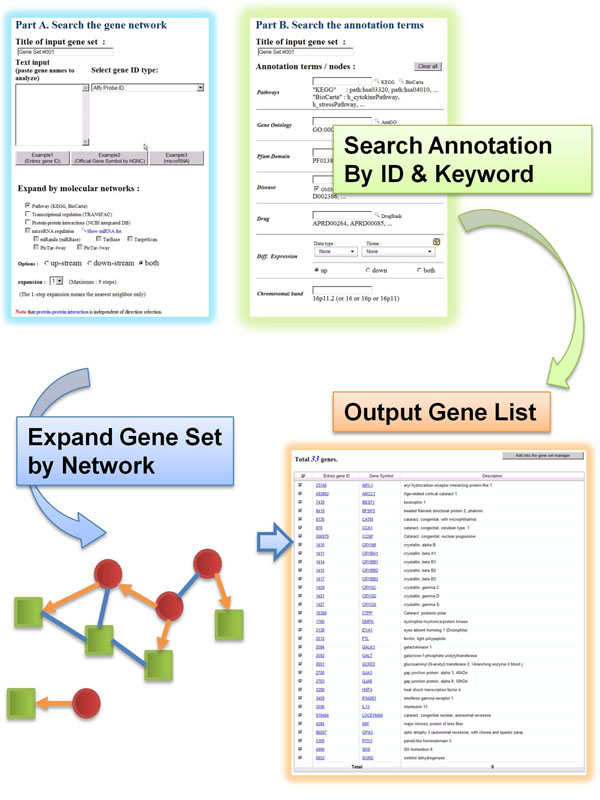
The gene set retrieval tool.

*Retrieval Tool* is for getting the member gene list of existing annotations and for expanding the existing gene sets via various types of biological networks. For example, miRNA target genes can be expanded via transcriptional regulatory network, and then by PPI network and so on (Figure 4). Gene set expansion via networks can be useful in combination with set operations e.g. intersection and difference in the *Manager Tool*, allowing a great freedom to navigate and combine the different aspects of existing knowledge. All the gene sets in GARNET is accessible by keyword or ID search and the member genes are listed in the resulting page (Figure 5).

## Conclusions

GARNET (gene annotation relationship network tools) is an integrative platform for diverse types of gene set analysis, where complex relationships among gene annotations can be easily explored with an intuitive network visualization tool.

## Availability and requirements

GARNET is available at http://garnet.isysbio.org/ and http://ercsb.ewha.ac.kr/garnet/.

## Competing interests

The authors do not have any competing interests.
